# An analysis of visual masking, with a defense of ‘Stopped Processing’

**DOI:** 10.2478/v10053-008-0014-3

**Published:** 2008-07-15

**Authors:** Adam Reeves

**Affiliations:** Dept. of Psychology, Northeastern University, Boston, USA

**Keywords:** masking, metacontrast, stopped processing

## Abstract

The use of a backward mask (a patterned mask which follows the target in time) to ‘stop the processing’ of the target illustrates an important application of masking – the study of the ‘microgenesis’ of visual perception, that is, visual processing over about the first one-fifth of a second. This paper provides evidence for stopped processing and some applications of this to object recognition and letter detection. The paper also discusses the notion of an ‘active filter’ which may help to account for Type-A masking but at best can only account for Type-B masking in part. I conclude that masking, while illuminating various areas of vision science, is under-utilized, perhaps because the theoretical justification for such masking is still uncertain, and perhaps because of the care needed to establish that the mask does indeed ‘stop’ processing.

## Introduction

Studies of visual masking include those which seek to explain masking, and those which utilize its existence for the sake of studying other visual processes. Masking arises when the report of a primary or ‘target’ stimulus is interfered with by a second or ‘masking’ stimulus. The term ‘backward’, in contrast to ‘forward’, refers to a mask which follows the target in time. Backward masking in general may be by flash (a bright, uniform second stimulus), by a non-overlapping pattern (‘meta-contrast’), or by a patterned mask which overlaps the target spatially (‘backward masking by pattern’). I will refer to this latter case as BM for succinctness. The use of BM to ‘stop the processing’ of the target illustrates an application of masking to study the visual processing of the target stimulus over the first one-fifth of a second.

I will first defend the notion that a carefully-chosen BM (or patterned backward mask) can indeed ‘stop the processing’ of the target, in Sperling’s (1963) phrase, by diverting resources away from the target to the mask, and leaving the representation of the target pattern in an early, incomplete form. Some authors have used the term ‘erasure’ (e.g., Schultz & Eriksen, 1977) to convey this idea, although this is often too strong a word. Several examples will then be given from already published work in which unexpected or theoretically interesting results have been obtained using this approach.

## Effective contrast

It was recognized early on that a patterned mask may reduce performance in identifying or detecting the target not by stopping its processing (Sperling, 1963, 1967) but rather by integrating with it, that is, by forming a composite representation in which features of the target are degraded (e.g., Eriksen & Hoffman, 1963). In this case adding a mask does little more than decrease the effective contrast of the target, so that the masking procedure merely complicates what could be studied more directly by lowering target contrast in no masking (NM) conditions. Indeed, to the extent that the BM falls within the critical duration for processing the target, it is hard to imagine how the mask would *not* act to reduce the effective target contrast. Yet the critical duration for luminance detection is typically about 35 ms, corresponding to the peak at 13 Hz in the modulation transfer function measured by flicker (see Reeves, 1996). Critical durations are longer for some other types of sensory information, such as color, but here we take the 35 ms as representative for passive integration at a sensory level for the usual luminance-defined targets presented at photopic levels to the light-adapted eye, a condition which is common in studies of masking by pattern. This duration is considerably less than the temporal span of masking, suggesting that masking is an active process of rejection, not just a passive loss of information due to temporal integration.

## Active filtering and information loss

An active process permits masking to be more than just passive temporal integration, in that information can be selected. Consistent with this idea, masking does *not* affect the quality of apparent motion signals even when these are brief enough to be within the time span of masking; such motion signals are critical for accurate vision and masking them might well disadvantage survival. An important implication of active filtering is that for masking to reflect any sort of useful (and fundamental) visual process, it should help *improve* the overall quality of information encoding. In principle, the loss of information implied by masking can only aid encoding if masking acts to filter out redundancy. Such redundancy must refer to local signals, those within the narrow spatial and temporal windows in which masking occurs. This idea applies naturally to Type A masking, in which masking is greatest when mask and target are simultaneous and decreases as they are separated in time. (Type B masking functions, in which maximum masking is delayed, are anomalous in this respect.) It is only very recently that natural scenes have been analyzed in sufficient detail for one to have any idea of how much local redundancy they contain. For example, Frazor and Geisler (2006) found that highly local image patches from images of foliage are redundant – that is, they correlate in luminance or in contrast value by more than *r* = 0.25 – if they are within 1 to 2 deg of visual angle of each other. This ‘correlation length’ varies with patch size, the type of scene, and the measure, being tighter for contrast than for luminance. No-one yet knows the correlation lengths over time, although head and eye movements will determine much of the variance, not just local motion signals, so further investigations are needed. Yet, it is to be hoped that the final outcome will permit formation of an ‘ideal masker’, one which will optimally attenuate or filter out redundant local signals in natural scenes and provide a bench-mark against which the measured spatial and temporal extents of masking can be compared. Such an analysis may provide the raison d’etre for masking which is currently lacking. The rather vague notion of an ‘active filter’ may then become a little more precise, as a filter that is tunable for the type of information (e.g. contrast, color, texture) that must be extracted from a local region of the image in order to perform a specified task.

## Channel specificity

A basic principle of any such filtering process is that for masking to occur, *mask and target must be processed by the same channel*. Interference with the target by distraction, for example, would not count as masking on this definition. Moreover, ‘object-substitution’ masking would count as a different (if very important) process, as argued in detail by Enns (2004). An elegant example of within-channel masking comes from research with simultaneous masking, in which it has been found that luminance increments which mask other luminance increments do not mask chromatic signals, and vice-versa (Cole, Stromeyer, & Kronauer, 1990). The lack of cross-masking shows that luminance and chromatic information are processed by separate channels, discounting the small facilitatory interactions also reported by these authors. As an example of the opposite kind of result, it might have been expected that the ‘On’ and ‘Off’ luminance pathways, which are thought to be physiologically distinct, would not show cross-masking; but they do (Kolers, 1962), a result which shows that the well-known ‘On’ and ‘Off’ luminance pathways must eventually run together.

The inference *from the absence of masking to the separation of channels* is not water-tight, as masking may be absent even within a channel under certain conditions. For example, metacontrast is absent at 50 ms SOA using rod-detected targets and cone-detected masks, a fact which suggested to Alpern (1965) that these pathways are independent, but masking is strong in these conditions at 70-150 ms SOAs, vitiating this conclusion (Reeves, 1986). Nevertheless, channel independence can be inferred if masking is absent over a sufficiently wide range of stimulus conditions, as defined by the psychophysics of the pathway under study. This inference concerning channel separation has been under-utilized in vision, being restricted to a few studies using simultaneous masking and virtually none using forward or backward masking by pattern. True, the details can be tricky; for example, the relative independence of luminance masking from color reported by Cole et al (1990) breaks down at very high contrasts (Mullen & Losada, 1994), perhaps because of divisive inhibition, but even so, greater exploitation of this principle could perhaps yield new lines of research.

## Integration or Interruption?

If it is presumed that masking reflects a within-channel filtering process, one can ask what type of filtering? Whether a patterned mask temporally integrates with or summates with the target (passive filtering), or rather stops the processing of the target (an example of active filtering), can be decided using a series of controls which compare perception in BM with perception in either FM (forward masking) or in no masking (NM) (Liss, 1968). In NM, the mask is turned off and the target is degraded by presenting it briefly or at low contrast. The need for such controls was emphasized by Eriksen and colleagues (see review by Shultz & Eriksen, 1977), who argued that backward masks generally operate by integration, given that the Type A functions found in FM and BM are often symmetrical, as predicted by temporal summation. This is typically the case for random noise masks. The channel hypothesis suggests that to reveal active filtering, however, it is necessary to use long-duration patterned masks that share features with the target, so that the feature-detectors in the channel essential for identifying the target are just those which are engaged by the mask, thus ensuring that the mask will divert processing from the target. By comparing noise and patterned masks presented either to the same eye as the target or to the other eye, Turvey (1973) was able to separate peripheral integration from central masking; only the latter shows evidence for stopped processing. Therefore the criticisms mounted by Eriksen and colleagues, while powerful enough to have limited enthusiasm for the ‘stopped processing’ technique, seem less than devastating.

**Figure 1.**
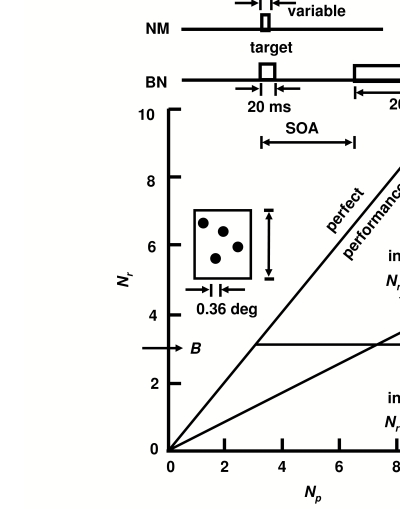
The number of disks reported (Nr) as a function of the number presented (Np) according to the integration model and the interruption models with the mask interrupting processing after B = 3 disks have been encoded. Above: temporal sequences in NM and in BM, where a blank ISI was introduced to vary the target-mask SOA. Insert: an example of a 4-disk target. The mask (not shown) was an 8x8 array of such disks.

**Figure 2.**
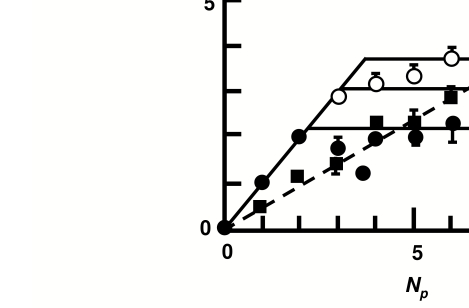
The mean number of disks reported as a function of the number presented (Np) in NM (black rectangles) and in BM at two SOAs, 30 ms (BM1: closed circles) and 50 ms (BM2: open circles). Values of B, the capacity limit in masking, were chosen to best-fit the data to the interruption predictions, separately in BM1 and BM2. Data follow the predictions of integration and interruption fairly closely, although averaging over individual participants smoothed the BM curves a little.

In our attempt to distinguish stopped processing from integration (Liss & Reeves, 1983), participants reported the number of black disks, from 0 to 10, presented at random locations within an 8-by-8 grid on a white screen. In NM, the disks were presented near-threshold by flashing them for just 2 or 3 ms to reduce their effective contrast. In BM the disks were presented at full contrast for 20 ms, but followed after a variable period by a 200 ms duration, patterned mask (Fig. 1, top). The mask was an 8 x 8 array of disks just slightly bigger than the target disks, located in the same positions as the target disks so that the masking was of the form ‘backward masking by pattern’ rather than ‘metacontrast’ in nature. The graph in Figure 1 presents the theoretical predictions of integration and interruption. Integration predicts that for low contrasts, the target integrates with the white field so the visibility of the disks is reduced. If the disks are far enough apart to eliminate lateral interactions, their chances of being seen are independent of one another. Thus the number of reported disks will be proportional to the number presented, at least until the number of items begins to exceed the short-term memory span. In contrast, the interruption theory predicts that the mask ‘stops processing’ when presented. In the (ideal) example plotted, the mask stops processing after 3 target disks have been encoded, so that performance is perfect for 0, 1, 2, and 3 disks, but no more than 3 disks are ever reported. The data for 6 subjects each clearly followed the integration prediction for NM, which accounted for 92% of the variance, and the interruption prediction for BM, which accounted for 90% of the variance (Fig. 2). In contrast, integration accounted for only 59% of the variance of the BM data, so the results clearly support the interruption model for BM over integration. This pattern of results was repeated for both strict and lax criteria for reporting a disk.

Critically, we also reasoned that if the BM really did stop processing, a subject given an SOA which limited him or her to 4 disks would not be able to tell the difference between a 6-disk target and a 10-disk target. In fact discrimination accuracy was 54%, hardly different from chance (50%). On the stopped processing account, the mask limited perception to just 4 disks, no matter whether 6 or 10 disks were presented. SOAs were short, 40 ms or 50 ms. Interruption theory also predicts that subjects should be able to tell 1-disk cards from 0-disk cards on every trial in BM at these short SOAs – and they could. How about NM? The stimulus duration was such that subjects reported 3 disks when 4 were presented. It is easy to show that an optimal observer who detects 3 of 4 (75%) of the disks, each being detected independently of the others, should report ‘10’ when seeing 7 or more disks, and otherwise ‘6’. Such an ideal observer will obtain 89% correct, a figure which is only slightly better than the 82% actually measured in NM. The difference between near chance in BM and near optimal in NM paints a vivid picture of the distinction between interruption and integration.

We also reasoned that if the mask truly stopped processing at 4 disks, then the subject should never be able to find all ten disks in a 10-disk display, because the participant would have no visual memory to carry over from trial to trial of the ‘unseen’ dots - they would be erased rather than simply too dim to justify reporting. In the experiment, subjects saw a particular 10-disk target cycled over and over for as long as they wanted. A faint 8x8 grid was added to the fixation field to aid dot-finding. Subjects were asked to pencil in the disks they saw on a similar report grid placed in front of them. In BM, there were no errors when the display contained one dot, but 3.6 errors (out of ten) when the display contained 10 disks, even after over 4 min of cycling. In fact, subjects eventually gave up trying to find all the disks; they could see a random sub-set of 3 or 4 on each trial, but they reported they could never see enough disks to fit the sub-sets together in the 8-by-8 grid. In contrast, subjects were quicker and made virtually no errors in NM; even though they could see only a few faint disks on each trial, they merely had to look around for a few exposures to piece together the entire target image.

## Scanning into the icon; or reading out from it ?

Some authors have suggested that ‘stopped processing’ implies that the BM acts to terminate the icon (Sperling, 1963), so that no more items can be read from it. However, the latencies for reporting the disks in Liss & Reeves indicated otherwise; after the subitizing region, in which latencies to report 0-3 disks increased at only 77 ms/disk, reporting additional disks was slow, taking on average 282 ms/disk in both NM and BM. Thus most disks were counted (or enumerated) well after both target and mask had disappeared! Participants informed us they reported from a visual memory of where the target disks had been, not from a continuing visual image. It seems that the mask curtailed *input* to the icon, not the persistence of visual memory.

The maximum number of disks reported increased with SOA at the rate of 20 ms per disk, a rate similar to Sperling’s (1963) estimate. The entire set of results can most easily be explained by a *serial scan* (Sperling, 1963) in which only one item is processed at a time, and each item is processed for 20 ms. When it appears, the mask stops further input to the icon by stopping this scan, but it does not degrade the visual memory of the icon, counter to the common interpretation. It is interesting that such fast scan rates, typical of feature search, are sometimes taken today as the hallmark of a noisy parallel process in which all items are processed simultaneously and independently (i.e., without mutual interference). Such an independent parallel model cannot handle these older data from backward masking. A model in which processing is initially parallel but terminates at different times on different items can, however, imitate a serial scan (Liss & Reeves, 1983).

## Rotate to recognize; an example of using stopped processing

De Caro and Reeves (2000) were concerned to test the ‘rotate to recognize’ theory of object recognition, in which mis-oriented objects are first mentally rotated before they can be matched to a canonical representation in long-term memory and identified. This theory had been supported by the longer reaction times obtained to identify mis-oriented objects; mean RT increases linearly with the degree of rotation away from the canonical orientation (Jolicoeuer, 1985, and many others). It is not obvious how this theory might explain the RT data, since if one had not already identified the object one would not know which way to rotate it; and if one rotated it the shorter way on half the trials and the longer way on the other half, only the variance of the RTs, not the mean, would change with the degree of mis-orientation. Therefore it seemed likely to us that the increase in mean RT represented a process subsequent to identification, such as double-checking the orientation of an already-recognized object, or perhaps a delay in the response due to the unexpected nature of the stimulus. To determine whether this was so, we followed a brief (16 ms) depiction of a common object with a blank ISI and then a 250 ms patterned mask. We designed the mask carefully with the aim of stopping further processing of the target object (see Haber, 1970).

**Figure 3.**
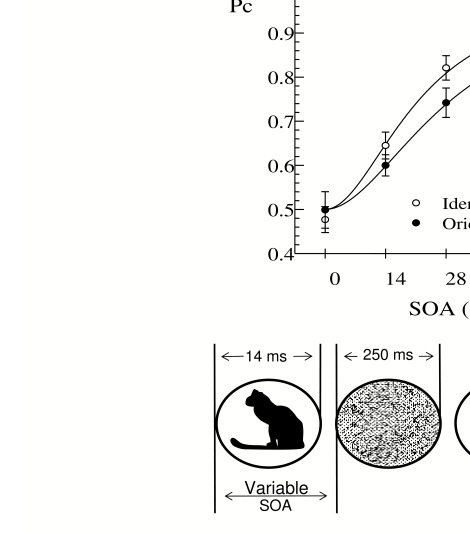
Top: The increase in accuracy (Pc) as a function of SOA, for reporting orientation and identity. Chance was 50% in both tasks. Identity is more accurate than orientation. Bottom: an illustrative trial, in which a stimulus (e.g. an upright rabbit) was shown for one 14 ms frame, and followed after a variable blank ISI by a 250 ms random-line mask (different on every trial). Participants’ knowledge of identity and orientation was probed after each trial.

**Figure 4.**
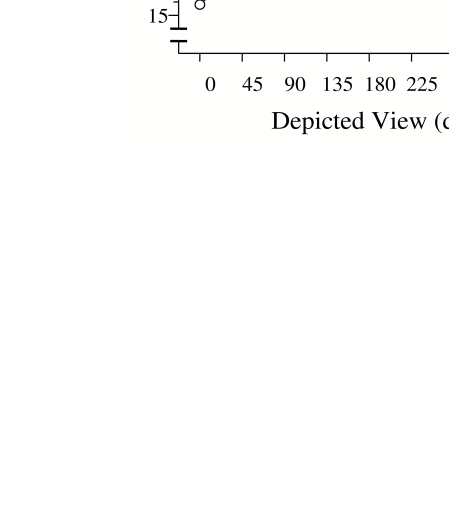
The critical SOA, that is, the (interpolated) SOA needed to reach 75% correct, as a function of the orientation of the object; 0 deg represents the canonical orientation, and other orientations represent mis-alignments. Critical SOAs are flat over object orientation for identification (open circles), indicating that participants did not ‘rotate to recognize’. However they peak for orientation judgments (closed circles), indicating that the objective orientation is harder to determine as the object is increasingly mis-aligned.

Participants reported whether the *identity* and *orientation* of the object matched a subsequent name probe (e.g., ‘rabbit’) and an orientation probe (an arrow); half the probes matched; half did not. Participants saw 96 line drawings of common objects, one on each trial, each being presented at one of several possible orientations. Not surprisingly, accuracy for reporting identity and for reporting orientation both increased with SOA from chance (50%) at SOA = 0 to better than 80% at SOA = 41 ms, but more important, at each SOA identity was more accurate than orientation (see Figure 3). Moreover, it was possible to determine whether there was any evidence of ‘mental rotation’ from plotting the SOA needed to attain 75% correct identification against stimulus orientation. Apart from slightly better performance at the canonical orientation, there was no evidence at all for mental rotation, as the critical SOA was flat across orientations (see Figure 3)(see Figure 4). However, the critical SOA for judging *orientation* did increase progressively with mis-orientation, being highest at 135 deg. We concluded that identity is indeed determined before orientation; that the time needed to obtain the identity of an object is independent of its orientation; and that the time needed to encode the orientation of an object increases the more it is mis-oriented. The important theoretical implication is that identity is obtained from mis-orientated depictions of common objects by a viewpoint invariant process (De Caro & Reeves, 2002). This is a common view (Marr, 1982), but not one that had been supported by reaction time data.

## Erasure without a physical mask

**Figure 5.**
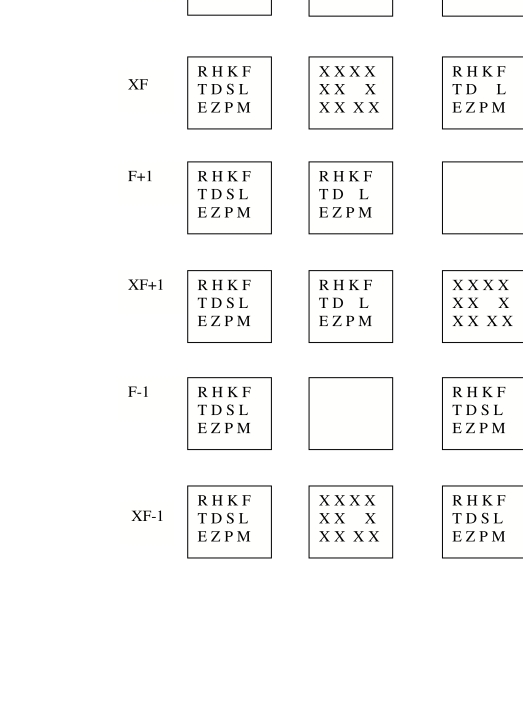
Participants saw a frame of 12 randomly-chosen letters of which one was removed to leave 11 behind (row 1; NF), or they saw the 12-letter and 11-letter frames with a blank white frame in between (row 2; F), or with a set of X’s between (row 3; X), or they saw other orderings of the displays (subsequent rows, e.g. F-1 or F+1) used to displace the blank white frame or the X frame by one position earlier or later, respectively, in the sequence. In some trials 4 or 6 letters were removed, not just one as shown.

Is it necessary to present a physical mask in order to ‘stop processing’? According to the general conception outlined here, it may not be; it is merely necessary to find some way in which processing can be diverted from the target before it is fully encoded. Here I take a leap and suggest that a ‘null stimulus’, if analyzed by the same channel as is analyzing the target, can pre-empt the target. By a null stimulus I mean something that turns off the feature detectors which are working on the target, without replacing the old information with new information. To illustrate, Charles Tijus and I (2004) presented a single frame of 12 black letters on a white screen. A random letter disappeared in the next frame, leaving 11 behind, and the participant had to identify it, the missing letter (Figure 5, row 1, the NF or No Frame condition). Even though both frames were only 16 ms long, this was possible on 81% of the trials. We then interposed a blank white frame between the 12 original letters and display of 11 letters (Figure 5, row 2; the F condition). Accuracy for reporting just one mising letter dropped to 24 %. Since the blank frame was white, homogeneous and identical in luminance to all the frames that preceded and all those that followed the display frames, there is no question of energetic masking or indeed of any other known type of masking. So, could the blank white frame have acted as a form of ‘mask’ at all? To answer this, we interposed a frame of 12 letter X’s instead of the blank white frame (Figure 5, row 3; the XF condition), and accuracy dropped to 30%. Thus the frame of X’s (which exactly replaced the 12 letters in the first display) was almost the equivalent of the blank frame in its deleterious effect. Since all stimuli were high-contrast, the X’s would normally be taken to act as a backward patterned mask. One would assume that the first display was backward masked by the X’s, so the participant would have little idea of the identity of the missing letter, which was only been presented in the first 12-letter display. On this logic, a blank white frame can, amazingly, also act as if it were a patterned backward mask.

**Figure 6.**
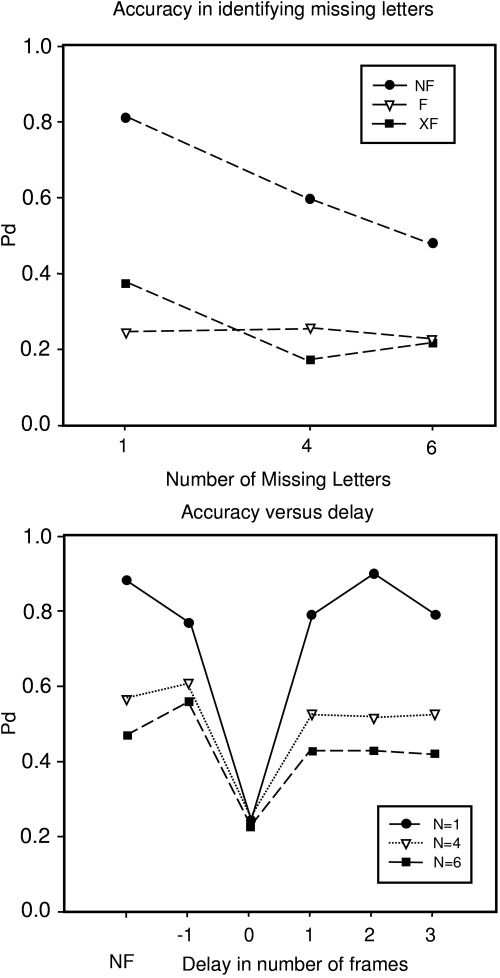
Top: Accuracy for reporting the missing letter(s) when there was no blank frame (NF), a blank frame (F), or twelve X’s (XF) in the positions of the letters. Accuracy was high in NF and degraded in F and XF, whether 1, 4, or 6 letters went missing (abscissa). Bottom: Accuracy as a function of the delay of the blank white frame or XF. Erasure only occurs if the blank white frame or X’s immediately follows the 12-letter display; the erasure effect is restricted to 16 ms or so.

We also tried reports of 4 and 6 missing letters; these were more difficult than reports of just 1 missing letter, but once again, accuracy dropped equally due to interposition of the blank white frame or the X’s. Figure 6, top, shows the full story; accuracy (Pd) is plotted against the number of missing letters in NF (black circle), F (open triangle), and XF (black squares).

If we varied the SOA, what would happen? We placed the blank white frame at various times before or after the second display, as illustrated in Fig. 5 (bottom four rows), and found that only when it immediately followed the 12-letter display did it act as a ‘mask’ (see results in Fig. 6, bottom). We therefore speculate that the reason for backward masking in this experiment is that the blank white frame acts to ‘reset’ the visual buffer containing the 12-letter display, and it does so because as a null stimulus, it is an informational mis-match to the letters which is just as severe a mis-match as a set of X’s. When only 1 of the 12 letters disappears, the two frames contain almost entirely congruent information and there is no reset, so visual memory is not erased, and the participant can recall the missing letter. Whether this form of rapid, almost instantaneous, informational masking really exists, and if so, how it is related to other forms of masking, remains to be seen; it is clearly distinct from object – substitution masking (e.g. Enns, 2004) in its time-course.

## Type-B curves in Metacontrast

Although the idea of an active filter may seem attractive for explaining backward masking, it cannot explain the Type-B curves obtained in metacontrast, in which a spatially non-overlapping mask has its maximum effect not at simultaneity but when delayed by 60-80 ms or so relative to the target. If local spatio-temporal correlations do indeed provide a reason (reduction of redundancy) for Type-A backward masking, it seems impossible for the same explanation to hold for Type-B data. However, acting on an idea of Neumann’s (1979), I had run various flanking bars experiments in which participants not only rated the visibility of the central target bar (the target), but also reported whether the flanking and central bars appeared to be simultaneous or successive (Reeves, 1982). Targets were presented on steady (photopic) adaptation fields. At central SOAs (60-120 ms), both types of trials were frequent enough for visibility in succession and in simultaneity to be traced out as a function of SOA. When the stimuli appeared to be successive, Type A masking resulted; target visibility increased monotonically with SOA; the flanks interfered less and less with the processing of the central target bar, the more that they were delayed. However, when target and mask were judged to be simultaneous, the inverse happened; target visibility *declined* with increasing SOA. (Only when the temporal order judgment was ignored and the data averaged over, did the familiar U-shaped curve emerge.) These results for flanking bars masking replicated those of Neumann (1979) for disk-ring masking, and also provided direct evidence against Kahneman’s ‘impossible motion’ account of metacontrast in that masking occurred even when the target and mask were judged to be simultaneous. One explanation for these results is that detection of the target is mediated by a slower channel (one that obeys a single-process explanation of masking) when simultaneity is judged, and by a different, faster channel (also single-process) when succession is judged. I rejected this idea, given that rods and cones feed into different, slow versus fast, post-receptoral channels, because the same pattern of results was obtained, albeit shifted on the SOA axis, when the wavelengths of the adaptation field, target, and mask ensured detection of the target by rods or cones and the mask by rods or cones (Reeves, 1986). [Note: target and mask luminance were fixed in Reeves (1982) but varied widely in Reeves (1986), lending generality to the results.] To explain his original results, Neumann (1979) had postulated two processes, implicitly acting within the same channel, with both processes working on each trial. One process favors temporal integration and the other temporal differentiation; the winner (implicitly) reflecting the dominant process and thus determining the temporal order judgment on each trial. If this is correct, as I believe (Reeves, 1982, 1986), then only the simultaneity data need a special functional explanation, as the succession data follow the type-A pattern. What might this be? 

Any possible explanation must also deal with the recent results of Francis (2005), who was unable to obtain evidence for the integration and differentiation processes in a 4-alternative disk-disk experiment with white targets and masks on a black field. In his data U-shaped curves were obtained whether the stimuli were judged simultaneous or successive; moreover, the curves overlapped, the result expected from a single-process view (Reeves, 1982). The reason for this discrepancy is as yet unclear, but it may be that light adaptation is required for the two processes to be revealed, although why this might be is not clear from Neumann’s (1979) two-process explanation. My current speculation follows from the obvious fact that the target and two masks form a single icon of three stimuli in the ‘simultaneity’ case. The monotonic loss of visibility that occurs as SOA increases in simultaneity trials (Reeves, 1982, 1986) could happen because at the moment the flankers (or outside rings) are assigned their visible contrast, the representation of the target has already begun to decay; the more so, the longer the wait to assign the target its visible contrast. At the same time, as the SOA increases, it became more and more likely that the central and flanking bars will be placed in successive ‘psychological moments’, or distinct temporal episodes, so the less likely it will be that the later-coming flankers will interfere with the central target. In Francis’s experiment, the field is black and hence target contrast is undefined; thus, target visibility is determined by brightness rather than by contrast. If so, and this is all speculation, some single-process explanation of metacontrast may be correct in the dark, but not in day-lit viewing conditions.

## Conclusions

Studies of visual masking have by and large been orphaned from those of vision per se; the emphasis in the masking literature has typically tuned inwards towards accounting for the intricate facts of masking, rather than outwards towards other areas of research in visual perception. Although simultaneous masking has been used in some cases to help identify separate channels, the various facts of masking do not seem to have leant themselves to use in other areas. One only needs to scan the literature on depth perception, say, or color vision, to see this. However, if the theoretical basis for masking can be established, the methods can be usefully applied to other fields. This point was illustrated in this paper by the still-controversial, but I believe, valid, use of a backward mask to ‘stop processing’, as suggested by Sperling (1963) so long ago. Another point of contact with the field in general, with its emphasis on the ideal detector, is to explain masking not just in terms of the underlying physiology but also in terms of its functional role. Here the masking field is in it infancy, and indeed the notion of a useful and active filtering process outlined above may yet turn out to be a ‘red herring’.
